# Conditions for sustainability of Academic Collaborative Centres for Public Health in the Netherlands: a mixed methods design

**DOI:** 10.1186/s12961-015-0026-7

**Published:** 2015-08-21

**Authors:** Maria WJ Jansen, Hans AM van Oers, Mizzi DR Middelweerd, Ien AM van de Goor, Dirk Ruwaard

**Affiliations:** Department of Health Services Research, School for Public Health and Primary Care (CAPHRI), Faculty of Health, Medicine and Life Sciences, Maastricht University, PO Box 616, 6200 MD Maastricht, The Netherlands; National Institute for Public Health and the Environment (RIVM), Bilthoven, The Netherlands; Tranzo Scientific Center for Care and Welfare, Tilburg School of Social and Behavioral Sciences, Tilburg University, Tilburg, The Netherlands; Academic Collaborative Center for Public Health, Public Health Service South-Limburg, PO Box 2022, 6160 HA Geleen, The Netherlands

**Keywords:** Collaborative networks in public health, Knowledge production Mode-2, Push and pull, Scientific and socially robust evidence

## Abstract

**Background:**

Contemporary research should increasingly be carried out in the context of application. Nowotny called this new form of knowledge production Mode-2. In line with Mode-2 knowledge production, the Dutch government in 2006 initiated the so-called Academic Collaborative Centres (ACC) for Public Health. The aim of these ACCs is to build a regional, sustainable knowledge-sharing network to deliver socially robust knowledge. The present study aims to highlight the enabling and constraining push and pull factors of these ACCs in order to assess whether the ACCs are able to build and strengthen a sustainable integrated organizational network between public health policy, practice, and research.

**Methods:**

Our empirical analysis builds on a mixed methods design. Quantitative data was derived from records of a survey sent to all 11 ACCs about personnel investments, number and nature of projects, and earning power. Qualitative data was derived from 21 in-depth interviews with stakeholders involved. The interviews were tape-recorded, transcribed, and manually coded as favourable or unfavourable pull or push factors.

**Results:**

The extra funding appeared to be the most enabling push factor. The networks secured external grants for about 150 short- and long-term Mode-2 knowledge production projects in the past years. Enabling pull factors improved, especially the number of policy-driven short-term research projects. Exchange agents were able to constructively deal with the constraining push factors, like university’s publication pressure and budget limitations. However, the constraining pull factors like local government’s involvement and their low demand for scientific evidence were difficult to overcome.

**Conclusions:**

A clear improvement of the organizational networks was noticed whereby the ACC’s were pushed rather than pulled. Efforts are needed to increase the demand for scientific and socially robust evidence from policymakers and to resolve the regime differences between the research and policy systems, in order to make the bidirectionality of the links sustainable.

## Background

In 2011, Nowotny, Scott, and Gibbons proposed a new form of knowledge production in ‘*Re-thinking Science: Knowledge and the Public in an Age of Uncertainty*’ [1]. The authors concluded that the line which formerly demarcated society from science is regularly being transgressed and that the resulting closer interaction between science and society signals the emergence of a new kind of science: contextualized or context-sensitive science. They called this new form of knowledge production Mode-2. Briefly, Mode-2 asks for ‘socially robust knowledge’ that is not only scientifically reliable but also involves societal actors outside academia to take societal needs and demands into account from a transdisciplinary perspective. Therefore, contemporary research should increasingly be carried out in the context of application, that is, problems should be formulated from the very beginning in dialogues among a large number of different actors and disciplines, taking their perspectives into account. This is generally true for all types of science, as problems in our society are very complicated. Tackling these complex problems requires multidisciplinary skills and expertise and open-ended chains to facilitate a dynamic multidirectional knowledge production process.

Contemporary issues in the field of public health are also often very complex, wicked problems [2] that require Mode-2 knowledge production. So far, knowledge is still mainly being produced by scientists working within their own scientific institutes. The institutional research system traditionally has no natural links with the public health policy and practice systems. The different systems each have their own regime and work as silos, making mutual alignments to produce Mode-2 knowledge a rare occasion [3]. In many countries, a joint procedure in which scientists, policymakers, practitioners, and citizens jointly seek suitable and context-sensitive answers to their questions is still rather uncommon [4–6]. Finding a new way of conducting context-sensitive scientific research and producing socially robust knowledge in the field of public health requires innovative procedures and research methodologies. An example of a context-insensitive intervention is the Human Papilloma Virus campaign in the Netherlands to prevent girls from getting cervical cancer. The campaign failed due to mainly medically-oriented vaccination messages. Although this campaign has been built on evidence from randomized controlled trials (RCTs) in which the context is controlled for, it failed to investigate social opinions and social media effects of the real-life context before introducing the campaign [7]. The RCT is still considered the ideal research method. However, knowledge based on the RCT method is no longer always suitable and can often not be valorised in society [8]. To mitigate these serious problems, we need context-sensitive science to produce knowledge that is valued by local and national policymakers for their deliberations on policy measures that are applicable in a specific context, e.g. real-life context. Knowledge production in public health should therefore be more tailored to what users of public health policy and practice need [9–13].

In recent years, innovative organizational formats have gradually been developed within the field of public health to facilitate a knowledge production that is more in line with the needs of society. Internationally, different initiatives are coming from public health policy, practice, and research institutes, and actors aim to build collaborative networks, agreeing to long-term commitment and time investment. Some examples from different countries are communities of practice [14], practice-based research networks [15], prevention research centres [16], knowledge brokers [17, 18], evidence-informed policy networks [19], the Pan American Health Organization [20, 21], collaborating centres for public health [22], and collaborations for leadership in applied health research and care [23]. All networks aim to produce knowledge that fits the demands of knowledge users, i.e. society, policymakers, practitioners, and citizens, and to carry out research in the context of application.

**Figure 1:**
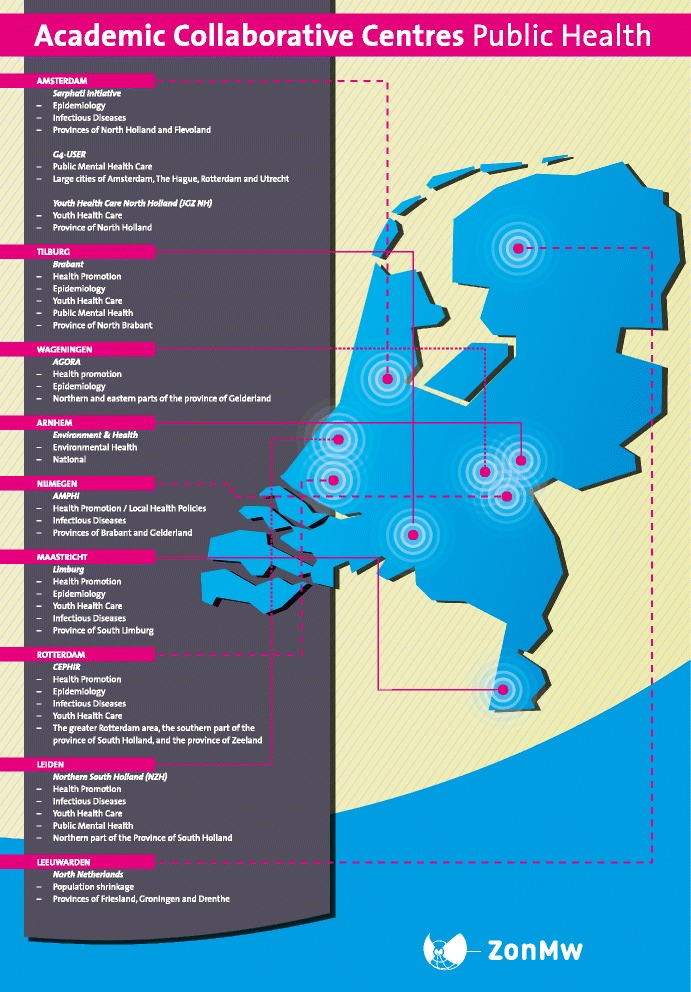
Overview of 11 Academic Collaborative Centres for Public Health in the Netherlands.

In the Netherlands, the Ministry of Health, Welfare and Sport has also initiated a similar type of collaborative network, in the form of the so-called Academic Collaborative Centres for Public Health (ACC) [24]. The Netherlands Organization for Health Research and Development (ZonMw) has been funding these ACCs since 2006. Eleven ACCs have been launched throughout the Netherlands (Figure 1).

Their aim is to build a regional sustainable knowledge production network, to invigorate the responsiveness of current public health research, and to work on context-sensitive and socially robust public health issues. An ACC consists of three different institutional partners: (i) local governments, which designs public health policy and decides on evidence-informed service supply and resource allocation; (ii) at least one regional Public Health Service (PHS), which monitors population health, initiates evidence-based community health promotion and protection and social action projects, and organizes service delivery with a wide range of public health professionals on behalf of local governments; and (iii) research institutes, which study determinants of population health and evaluate processes and assess the effectiveness and cost-benefit ratios of practice-based interventions or policy measures. The ACCs aim to structurally strengthen and anchor demand-driven research activities in the field of public health practice and policy. The PHSs were given the chair position in the ACCs, which was a requirement to receive funding. Because the PHS is formally controlled by the local government, ZonMw argued that the linking-pin between local policymakers and researchers could obviously best be positioned within the PHS, thereby putting the PHSs in the exchange agent position, in terms of Roger’s theory of diffusion of innovations [25]. Such exchange agents facilitate researchers in responding to the problems, questions, and demands of the local governments. Through continuous dialogue initiated by the exchange agent, knowledge users (i.e. policymakers, practitioners, and citizens) and knowledge producers (i.e. researchers) aim to jointly conduct practice-based research projects in the context of application.

The ACCs have been receiving financial support for 8 years. Investments by ZonMw amounted to €14.3 million ($19.1 million) in 2006–2010 and €16.3 million ($21.8 million) in 2010–2014 to build and ultimately secure the continued existence of 11 ACCs. A midterm evaluation in 2010 showed that the ACCs were able to build an integrated network structure where science, policy, and practice jointly produce public health knowledge [26, 27]. The rationale for creating a win-win situation for all partners stems from the challenge to overcome the difference in institutional accountabilities between knowledge users and knowledge producers. These accountabilities refer to scientists’ endeavour to publish in high impact journals; public health professionals’ efforts to organize the short-term practical application of interventions; and policymakers’ task to formulate policy proposals that are incremental so they are accepted by the public and by politics [28–31]. These different accountabilities may threaten the co-evolutionary processes that knowledge users and producers are engaged in within the ACCs, and may thus threaten the sustainability of the ACCs, the ultimate aim of this ZonMw-funded program on behalf of the Dutch government.

The midterm evaluation also revealed that the network structure had resulted in quite a large number of funded 4-year PhD studies. However, local governments could not be sufficiently involved and their interest in the PhD studies slowly faded during the 4-year period with regards to policymakers’ commitment to participating in the PhD trajectories [26]. PhD studies lost their attractiveness because submission to international peer-reviewed journals was often prioritized by the research system, while applicability of results in policy and practice was hampered by insufficient investments in bidirectional dialogues. This resulted in an imbalance in terms of win-win situations, especially for the policy and practice systems, since they did not obtain the answers that society needed nor practical solutions to their problems, as had been expected. In allocating funding for the second grant period, ZonMw therefore demanded that short-term studies based on user-initiated policy questions (taking a maximum of 6 months) should be added in order to strengthen cross-domain interactions between users and producers. It was assumed that short-term research studies elicited by local policymakers’ questions would produce knowledge relevant to society, and specific research evidence allowing timely application in policymaking. ZonMw argued that this would lead to a better win-win balance, which was supposed to be required to continue the ACCs once the funding ended, in December 2014.

We wanted to know the enabling and constraining push and pull factors in order to assess whether the ACCs are able to build and strengthen a sustainable integrated organizational network between public health policy, practice, and research.

### Push and pull theory

To answer our research question we used the theory of push and pull factors [32]. The concept of push and pull factors was originally devised in the context of distributing and selling products on the (commercial) private market based on supply and demand. We used the market principles because demand and supply are essential conditions for the sustainability of ACCs: can the ACCs sufficiently accommodate demand and supply in contemporary public health knowledge and thus be sustainable in the long term? The World Health Organization (WHO) previously attempted to apply these supply and demand principles to ‘better sell public health products’ [21]. A well balanced demand and supply system will strengthen the desired quality improvement on the public health market, according the WHO. Linkage activities between the producers’ and users’ side, such as long-term partnerships, exchange agents, or knowledge brokers, might reinforce the use and the production of public health research for policy and practice purposes. The WHO distinguished enabling and constraining push factors relating to the knowledge producers (supply) and pull factors relating to the knowledge users (demand) (Table 1). This distinction certainly does not mean that knowledge is produced by suppliers in a technocratic, linear way, to be subsequently translated for or distributed to users. In principle, Mode-2 knowledge production is a co-evolutionary cyclical process in which problems are formulated by both users and suppliers in an ongoing dialogue from the very start. We only use the push and pull factors to show whether temptation strategies of the exchange agents, e.g. discussions about the specific meaning of socially robust evidence in a community, can reinforce these factors, as they do in marketing. Mode-2 knowledge production can be pushed by attractive domestic conditions, e.g. when there is prominent support for an evidence-informed policy approach by senior researchers and a local government that supports this approach. It can, however, also be pushed by unfavourable conditions, e.g. when the problems are so complex that it is hard to find evidence. Similarly, Mode-2 knowledge production can be pulled by favourable conditions, e.g. when tacit or lay knowledge is highly valued in addition to scientific research knowledge and local policymakers commission participatory research to make science applicable to subgroups or a specific context [33]. However, Mode-2 knowledge production can also be pulled by unfavourable conditions, e.g. when politicians suggest they are awaiting the results of a more comprehensive, more evidence-based research report, just to win time. Push and pull factors may either be compatible with or clash with accountability aspects in each of the institutions. For instance, research-driven agendas aiming to produce publications in high impact journals for accountability reasons are a constraining push factor for policy and practice in Mode-2 knowledge production [34], while a high demand for scientific evidence to be used in incremental policy changes is an enabling pull factor for research, for reasons of accountability.

**Table 1:** **Modified from [** 27 **], Greenhalgh and Wiering [** 35 **], and World Health Organization [** 21 **]**

**Factors in mode-2 knowledge production between science, policy and practice**		
	**Enabling**	**Constraining**
Push factors/supply side	• Donor/funding agencies’ support for knowledge coproduction	• Evidence too complex
	• Availability of evidence	• Research-driven agendas related to publication in high-impact journals
	• Credible knowledge brokers and opinion leaders	• High cost of producing
	• Appropriate packaging in ‘evidence-based actionable messages’	• Packaging and distributing evidence too prohibitive
		• Poor local access to relevant evidence
Pull factors/demand side	• Problem-based evidence, user-initiated policy questions and tacit knowledge	• Financial reasons for not acting on evidence
	• Local knowledge champions	• Low demand for scientific evidence by policymakers
	• Political support for implementation of particular research evidence	• ‘Paradigm differences’ between researchers, policymakers, and practitioners
	• Strategic presence of social actors in local decision-making bodies (social participation)	
Exchange factors/exchange agent’s role	• Education of and dialogues with users and media regarding high-impact stories on the use of knowledge	• Lack of interactive communication between producers and users of scientific evidence
	• Innovative ways of knowledge sharing, esp. tacit knowledge and the community	• Lack of knowledge sharing, especially with policymakers

We answered our research question by analysing the organizational networks between public health policy, practice, and research in terms of enabling and constraining push and pull factors with respect to the institutional accountabilities of knowledge users and producers. Next, we analyzed the conditions necessary to make this organizational network sustainable in terms of enabling and constraining push and pull factors in the eyes of the main initiators. We explicitly pay attention to the role of the exchange agent, as a win-win balance in the knowledge production and utilization process can be moderated by such exchange agents [25, 36, 37].

## Methods

We aimed to obtain a more thorough understanding of the enabling and constraining push, pull, and exchange factors of the ACCs by using a mixed methods design, collecting data between September 2013 and March 2014. Quantitative data was derived from records of a survey sent to all 11 ACCs, asking the coordinators about the number and nature of projects (number of qualitative, quantitative, and mixed methods designs), their investment in the collaborative centre in terms of full-time equivalent (0.5–0.9, 1–1.4, 1.5–2, >2 fte) staff, the return on ZonMw investments (extra external grants secured by appointed ACC personnel, expressed in euros), website information about the projects (website, yes/no; up to date, yes/no; newsletters, yes/no; meetings, yes/no; project information, yes/no) and network formation of each ACC (collaboration structure). Nine of the 11 surveys were correctly filled in, one was partly filled in, all activities and information were categorized and counted. Qualitative data was derived from 21 in-depth interviews with all ACC coordinators in their role as exchange agents (n = 11 out of 11, code EA), program participants of the funding organization ZonMw because of their role as financier on behalf of the national government (n = 2 out of 10; selection criteria: 1 vice-chair and 1 member of ZonMw commission ACC-program; code Z), board members of the Association of Dutch PHSs because of their role in advocating the relevance of ACCs (n = 3 out of 25; criteria: board member and actively supporting the ACC; code A), and civil servants using short-term research project results (n = 5 civil servants out of 11; criteria: personally involved in short-term research according to coordinator; code C). Respondents were purposely selected based on the described selection criteria. All respondents agreed to participate. All the in-depth interviews were held by an independent researcher who was not involved in any ACC. Interviews focused on the enabling and constraining push and pull factors (Table 1) and whether the exchange agents were able to make these factors compatible with institutional accountabilities or to get compromises accepted. The interviews were tape-recorded and had an open character. When interesting comments were made on a particular pull or push factor or the exchange agent’s role, the researcher probed the participant for more details. Interviews lasted between 20 and 150 minutes. Transcripts were used by authors MM and MJ to manually code the push, pull, and exchange factors [38]. Their interpretation led to the selection of relevant parts of the interviews in order to search for consensus about enabling and constraining push and pull factors, and the role of the exchange agent to mitigate imbalances between the users and producers. The results were ranked according to the relative degree of importance, i.e. the frequency of comments by interviewees. Only after MM and MJ had reached full consensus about the interpretation, did we present our findings (as a member check) to the 11 ACC coordinators, who did not have any comments on the analysis or its results. Quotes from the interviews are used below to reflect on potential enabling and constraining factors relating to suppliers, users, and the exchange agents. All these together served as the empirical data sources for the present manuscript.

## Results

### Enabling push factors

#### Funding

The national funding by ZonMw during 2006–2014 was considered by the respondents to be the most important enabling push factor, both for the coordinating tasks and the research projects. Without this grant, the ACCs could never have achieved their present status.

“*The grant acted as a catalyst, and hugely speeded things up.*” (EA1)

With this grant, all 11 ACCs were able to build an infrastructure for knowledge production consisting of a coordinator (with a double appointment at a university and a PHS, with coordinating tasks for 0.2 to 1.0 fte), a small working group of PHS professionals that initiates new research projects (ranging from 0.5 to 5 fte), a steering committee of PHS directors and public health professors, and a contractual agreement about the network formation signed by the boards of the PHSs on behalf of the local governments in the regions they cater for and the universities. The contractual agreement explicitly defines the administrative support and commitment. Most ACCs consist of one PHS which takes the lead, sometimes in collaboration with other PHSs, and a nearby university, covering a catchment area of 600,000 to 1.5 million inhabitants (Figure 1).

#### University’s role for society

The interviewees reported that the university had an important role in the production of evidence that is both scientifically sound and context-sensitive. Without the university, an ACC cannot exist. The collaboration between universities, PHSs, and municipal authorities is considered to be important for future public health innovations.

“*The PHSs have come a long way, and are finally producing excellent results. At first they had to get used to the red tape involved in submitting research proposals, but we now understand why that’s necessary. The PHS has something to offer that the university can’t provide, and the university has something that the PHS doesn’t have.*” (Z1)

#### Special professorships

Enabling push factors are the professorships by special appointment in particular disciplines associated with the ACCs’ societally relevant public health topics, such as infectious disease prevention, prevention in primary care, youth health promotion, population health, and healthy cities. The ACCs have stimulated the creation of these professorships to strengthen societally relevant (and context-specific) public health research. ACC coordinators experience sufficient support from academia to discuss innovative research methods beyond the commonly used context-independent RCTs. In some ACCs, the university’s faculties of Social Sciences and Administration have joined in, as well as those of Medicine and Health Promotion, providing new opportunities for innovative qualitative and quantitative research methods that better fit the exploration of ‘fuzzy’ or ‘wicked’ public health problems.

#### Tacit knowledge utilization

Gradually, tacit knowledge among citizens is being valued more, and researchers are becoming aware that useful evidence must be triangulated with local knowledge to enable it to be applied in practice.

“*The collaboration with other parties, including officials and citizens, is very important for many research projects. For instance, if you want to improve school health policies, you need the experiences that pupils, parents, teachers, and school boards can provide. You can obtain this information through in-depth interviews, discussions, an understanding of contextual preconditions and the local context, and of course hard facts. And all this should be considered not only from the health care perspective, but also from that of the children’s perception of their world, in a bottom-up process. That way, the qualitative and quantitative research methods can reinforce each other*.” (EA9)

#### Post-academic education

Push factors were reinforced by additional activities in higher education. Seven ACCs systematically contribute to post-academic education, i.e. master classes for policy, practice, or research professionals to upgrade the research skills of their ACC team. In addition, they support educational courses in academia for Master students to attract young students for research in practice.

“*Most people have been taught these research methods at some stage, but if you haven’t used them for a long time, you tend to forget them. You think: How exactly did that work again? So then refresher courses in the form of short master classes are very useful. I think we’ve managed to get that set up pretty well in recent years.*” (EA11)

#### PhD research grants

Enabling push factors are the grants for PhD research projects. The number of long-term research projects, mostly in the context of PhD positions, has currently risen to approximately 140 (based on 8 of the 11 survey forms completed by ACCs; ranging from 3 to 53 projects, with a mean of 17.5 per ACC). Earning power in terms of external research grants (excluding the €30.6 million ZonMw grant) amounted to approximately €25–30 million (US$33–40 million) (based on 8 of the 11 ACCs, ranging from €100,000 to €7.5 million, with a mean of €3.5 million per ACC).

“*The minister has had a fantastic return for a modest investment: a wonderful network and wonderful projects.*” (Z2)

#### Packaging of evidence-based actionable messages in Dutch

Thanks to the collaboration, the packaging of evidence-based actionable messages has improved. Project results are summarized in fact sheet formats. Most ACCs organize expert meetings and joint learning platforms to share knowledge and discuss knowledge application. All ACCs release public-friendly newsletters to inform all stakeholders and give them access to research results. Most websites show summaries in Dutch of PhD research, to make these findings accessible to the lay public. In addition to scientific publications in high impact journals, ACCs stimulate publications in news media and Dutch journals to discuss applicability and feasibility for end-users (Table 2).

**Table 2:** **Results of the study**

**Factors in mode-2 knowledge production in 11 Dutch Academic Collaborative Centres for Public Health (ACCs)**		
	**Enabling**	**Constraining**
Push factors/supply side	1. ZonMw funding to build an infrastructure for knowledge production (coordinator, steering committee, contractual agreement, working groups)	1. Perceived pressure of university to publish in high impact journals
	2. University’s role in the production of context-sensitive evidence	2. University’s requirement for PhD projects
	3. Professorships by special appointment	3. Difficulty to find external grants for policy-initiated or practice-based research
	4. Tacit knowledge among citizens is being valued more by researchers	4. Unwillingness of Public Health Service (PHS) directors to really advocate ACC
	5. Post-academic education	
	6. Grants for PhD research projects	
	7. Packaging of evidence-based actionable messages in Dutch	
Pull factors Demand side	1. Short-term policy-driven research projects	1. Limited budget availability for infrastructure, especially for coordinator
	2. Local Alderman for Public Health acting as ACC champion	2. Limited involvement of local government
	3. Publications in public friendly Dutch journals	3. Low demand for scientific evidence by policymakers
Exchange factors	Interactive communication and indispensable linking-pin function with passionate attitude of coordinator	Limited influence of coordinator on decision-making process of local authorities and PHS directors

### Enabling pull factors

#### Short-term policy driven research projects

Enabling pull factors that were strengthened by ZonMw in 2010 relate to the short-term policy-driven research projects, whose number has risen from 10 to 160 (based on 9 of the 11 completed survey forms, with a mean of 17.7 projects per ACC, ranging from 3 to 26). The interviewees, especially those from the Dutch PHS Association and the civil servants, considered the short-term research projects to be a very important impetus for policy-initiated research. These short-term projects fit the needs of local governments, and the problem-based and value-driven nature of policymaking.

“*The perception study in the village of Beuningen is a good example of a short-term study. We examined how the local community in the area adjoining the motorway perceives the widening of the motorway. Although the decision to widen the road was made by the national government, some measures can be taken at local level. The decision-making process in the municipal council took the residents’ views seriously, and discussed how the impact on the residents could be alleviated. The short-term study contributes to well-founded decision-making and is appreciated by the municipal authorities in the area.*” (C3)

All interviewees confirmed that the growing number of these short-term project leads to intensified relations with local authorities and greater visibility of the ACC. Gradually, more civil servants have approached the ACC with policy-driven research questions. In some ACCs, Master students from the university conduct the research in real-life settings or systematically review existing knowledge, synthesize it and transform it into context-sensitive policy advice, free of charge. In other ACCs, the municipal authorities have to pay, as trained PHS professionals are commissioned to do the research. In general, the short-term research projects are interesting for academia when they can be valued in terms of educational credits, though not as scientific performance in terms of publications, because the results are generally not sufficiently scientifically profound.

“*The scientific quality of the* Klein maar Fijn *(Small but Beautiful) study is at the level of Master students, so it can be used very well as a Masters Degree project. The student learns how to apply knowledge, in many cases existing knowledge, in the local context. That makes it useful to both the municipality and the university. So you continually have to look for criteria to justify it to the individual parties.*” (EA8)

#### Local actors as champion

In some municipalities, enabling pull factors have emerged, e.g. a local Alderman for Public Health acting as a strong champion of the ACC. Interviewees considered these local advocates to be very supportive to the ACC because their support may affect decisions on the commissions for policy-driven research proposals to support evidence-informed policymaking.

“*Lack of support by the various parties is no longer a limiting factor; there’s enough of that from all parties. The local authorities are absolutely enthusiastic about what we do, including the municipal executive. They often mention that; they often refer to the ACC and what it is doing for them. They’re very much aware of that. We are thoroughly integrated in the PHS organization as well as in the university organization: there are several professors that have embraced this approach and support it. These are solid links.*” (EA4)

### Enabling exchange factors

All PHS coordinators have tried to strengthen cross-domain interactions between knowledge users and producers by building a website and improving their communication strategies. Nowadays, it is clearly recognized that a largely passive communication strategy, focusing on diffusion through scientific journals, is not sufficient to make knowledge applicable and useful for policy and practice. All 21 interviewees considered the role of coordinators as exchange agents to be indispensable. The coordinators’ drive makes them a very relevant linking-pin between the three systems, i.e. the university, the local government, and public health workers. Coordinators regard themselves as pioneers, who are very eager to use all opportunities to bring the different paradigms and accountabilities together. They are enthusiastic about the many new projects that have emerged from bottom-up initiatives in local communities, and act as social entrepreneurs.

“*Yeah, I’m absolutely passionate about this. I’ve been doing it for years and I hugely enjoy it. I couldn’t think of a better job. There’s so much left to discover and to develop.*” (EA4)

### Constraining push factors

#### Publication in high impact journals

Most coordinators consider the pressure to publish to be a constraining push factor. Although scientists increasingly seem to recognize the importance of context-sensitive research, as it determines the feasibility and effectiveness of public health activities in a real-life setting, it is still difficult to avoid this publication pressure. Besides, context-sensitive PhD research projects do not easily result in scientific publications, as international journals, especially the high impact ones, are not interested in these.

#### University’s requirement for PhD projects

The interviewees confirm the university’s requirements for publications and PhD projects. If, for instance, practice-based research does not result in publications, the interviewees experience a loss of interest on the part of the university, or the research is only marginally supported.

“*What I find a bit problematic about this collaborative centre from the point of view of the universities is that the first three years have not seen a lot of research in the field of public mental health that has led to publications. A lot of short-term studies, very interesting and also very valuable for policy and practice, but it has yielded relatively few academic publications. That is a bit of a problem with regard to keeping the universities involved, as that’s what they are expected to produce.*” (EA5)

#### Depending on external grants

The sustainability of the ACC research projects mainly depends on external grants. Financial support from academia, local government, or PHSs is not regarded as an option because each institute has its regular tasks and legal obligations that cannot be reduced to create room for ACC-initiated research. All ACCs consider it very difficult to find grants for policy-initiated or practice-based research projects in the competitive world of funding.

“*Traditionally, it’s the universities that are on the committees that have to decide on the research proposals the ACCs submit. Those topics* [i.e. the practice-oriented ones] *are often rejected, as ‘this is too practical, it won’t produce any scientific results, it’s not theoretical, it’s not longitudinal research, it’s not an RCT.’ They assess it using the traditional scientific standards, and so you won’t manage to get support for a topic that’s mostly interesting and relevant to practice and to local governments*.” (EA8)

#### PHS directors’ attitude to advocate ACC

A constraining push factor relates to the attitude and opportunities of PHS directors. PHS directors should stimulate strategic stakeholders to support the ACC, but the interviewees doubted the power and willingness of the director to seriously and continually advocate the ACC, because there are many other, probably more important or controversial, issues that need their attention.

“*You find that what the directors do in their own PHS differs from what they say they’re going to do in national discussions. Sometimes they just don’t have enough opportunities to realize what they would really like to do. Some directors are very subservient to the local authorities, and they don’t have enough power to push things through, as it’s the PHS itself that should try to make things happen.*” (A2)

### Constraining pull factors

#### Limited budget from local government

A strong constraining pull factor is the budget available from the users’ side, i.e. the local government. Every year PHS and local authorities make agreements on products to be delivered by the PHS. This means that budgets and capacities are fixed. To make investments on the development of an ACC requires negotiations with local authorities, unless this is permitted by available PHS budgets. However, PHS managers lack sufficient budget to build capacity and some ACC professionals from the PHSs have to fulfil their tasks within no more than 0.2 fte.

“*I notice how easily the management signs the collaboration agreement, as if they’re saying ‘Of course we’re going to do that’. And then what? They don’t think about the consequences, in term of allocating at least one day a week for a few people to do that work, and setting it down in a plan of action. Concentrating on intentions not consequences. That’s not going to work. So you enthusiastically announce the collaborative centre and assign certain tasks to people, which they feel are being imposed on them, but you don’t give them the time to do them.*” (EA1)

About half of the ACCs experience problems securing sustainable funding for the network, especially when PHSs are unable to incorporate the role of a coordinator in their regular annual budget. The interviewees stressed the need for local research and development priorities at the PHSs to continue the ACCs. One ACC had acquired research and development funding that had already been decided upon by the city council about 10 years ago, even before the ACCs were established, to stimulate PHS innovation. This ACC ranks highest in terms of both short- and long-term projects and earning power.

#### Limited involvement of local government

A constraining pull factor concerns the involvement of local governments. Despite eight years of improving communications and building knowledge platforms, the ACCs still lack the necessary profile, especially among local authorities.

“*I think that if you ask people at the PHS and in the local government what the ACC is and what it could do for them, you’ll see a lot of puzzled faces.*” (EA9)

#### Low demand for evidence

Constraining pull factors also relate to political deliberations and implementation preferences that lack a systematic overview of existing knowledge and alternative approaches. The interviewees mentioned ignorance on the part of policymakers, lack of visibility of the ACC, and financial constraints as reasons why evidence is not acted upon in the political discourse. Local advocates are still too few to have a meaningful impact on evidence-informed policy making. All interviewees agreed that it is ultimately the locally responsible policymakers who have to decide whether they allocate extra research budget to look for evidence to support policy measures in the context of applicability. The PHS can stimulate this but it is the policymakers who have the final say.

“*The main question is whether the PHS is given enough support by the officials. Do the officials, which mostly means the municipal executive, understand the importance and the added value of additional research for their municipality, and are they willing to invest money and staff?*” (EA3)

### Constraining exchange factors

The PHS coordinators emphasized that they have a meaningful role to play in initiating and supervising research projects, but their strategies as exchange agents with respect to the sustainability of the ACC network are limited. They feel they have limited influence on the decision-making process of local authorities and PHS directors when it comes to sustaining the ACCs. The political context and municipal budget cuts are complicating factors that are difficult to address. All interviewees expected that the debate about sustainability would continue at national and local levels in the coming years, as there are no simple solutions.

## Discussion

In this study, we investigated the necessary conditions for the sustainability of networks between public health policy, practice, and research, in terms of enabling and constraining push and pull factors, with users on the demand side and producers on the supply side of knowledge production. Such links are needed in the Mode-2 knowledge production process to generate socially robust and context-sensitive public health knowledge. The results show that the work of the ACCs leads to a clear improvement of the organizational network. Many practice-based and policy-driven short- and long-term research projects have been initiated by the producers’ side. The network succeeded in obtaining external grants for about 150 short- and long-term projects, amounting to about 25 to 30 million euros. Joint knowledge-development groups consisting of researchers, practitioners, and policymakers were formed to combine research evidence with local context-sensitive information, in order to find the best approach to develop local public health policy. Generally speaking, the groups were able to overcome the constraining push factors, such as the complexity of the evidence, the production costs, the packaging and distribution of evidence, and the access to relevant literature. By far, the most enabling push factor was the ZonMw funding. The enabling pull factors relating to the users also improved during the funding period: strategic stakeholders positioned themselves as local champions and the number of policy-driven short-term research projects grew substantially. However, constraining pull factors, such as the low demand for scientific evidence from policymakers, political or financial restrictions, the regime differences between the research system and the policy system, and the lack of visibility of the ACCs, limited the bidirectionality of the links. The results show that the ACC seemed to be more pushed than pulled, because many more activities were developed at the supply side compared to the demand side.

With regard to the conditions necessary for sustainability, we analyzed whether the push and pull factors interfered with the different accountabilities. We argued that, if institutional accountabilities could be met in a win-win balance between the partners, the organizational network would be sustained. The results show that long-term research projects were pushed by the universities because they resulted in publications in high impact journals, but these were not pulled by the municipal authorities. Municipal authorities demanded short-term research projects, as they contributed to timely and context-sensitive policy adjustments, and these short projects were only pushed by the universities when they could function as internships for their Masters programs. Only a few ACCs were really successful in combining long- and short-term research projects, and in making and keeping both universities and local governments enthusiastic.

ACC coordinators were able to influence the push factors, but felt that the pull factors were mostly in the hands of local political stakeholders such as PHS directors and local authorities. Despite the double appointments of the ACC coordinators at universities and PHSs, their position seemed more strongly embedded in the knowledge-producer system than in the knowledge-user system. An important constraining push and pull factor was related to budgets. The ACC coordinators argued that this factor, relating to both the user and supplier sides, was difficult to change, due to municipal budget cuts. Despite the fact that all PHS directors promised to prioritize public health research, only a few really succeeded in earmarking budget within the existing annual budget of their PHS. This raises questions about the actual willingness to innovate and about PHS directors’ ability to overcome managerial impediments and change routines into Mode-2 public health knowledge production.

Our findings are comparable to those of other studies in this field that also found stronger push rather than pull strategies, a low demand for scientific evidence by policymakers, and a strong institutional pressure from the university partner to produce high quality academic publications [15, 16, 22, 23, 34]. Greenhalgh and Wiering [35], Kok and Schuit [3], Green and Mercer [39], and Wehrens et al. [40, 41] showed the complexity of managing the different accountabilities and the inherent enabling and constraining push and pull factors. Nowotny et al. [1] expressed the need for a paradigm shift. Kuhn, the founding father of paradigm shifts, explained that paradigm shifts only occur when scientists encounter anomalies [42]. Such anomalies will only occur, however, when the different systems, each with their own regime, collapse or change radically. Although some small cracks in the system are currently appearing, the system change will, in all probability, be incremental and take more time [43].

A strength of this study is our unique exploration of push and pull factors to accommodate demand and supply in public health knowledge. The multiple-method design we used provided more compelling and robust evidence than a single method could have done. Nevertheless, our study did have some limitations. First, the number of interviewees was small and might not be representative of the entire group of ACC-stakeholders. There were relatively more coordinators. This raises the question of whether the results are a reflection of push and pull factors of stakeholders in general or a reflection of push and pull factors experienced by the coordinators. Besides, the interpretation of the interviews was only fed back to the coordinators, and not to the other interviewees, for practical reasons. A member check for the latter group is thus missing. We tried to prevent interpretation bias by using two interpreters who independently coded the interviews. The second limitation might be the fact that most local authorities were represented by their PHS in the ACC. The real voice of local authorities might thus have been insufficiently expressed, except for the policy-driven short-term research projects. The results of our study should therefore be interpreted cautiously and need to be substantiated with further empirical data. The fact that we found similar results in previous empirical studies strengthens generalizability.

Based on our findings, we recommend putting more emphasis on strengthening the pull factors. The users, i.e. policymakers and practitioners, should become aware of the need for evidence-based and evidence-informed solutions to contemporary public health problems. Local governments will be increasingly confronted with complex public health problems as the meaning of health gradually shifts toward a dynamic concept related to the ability to adapt and to self-manage, in the face of the challenges posed by the community [44]. Further, more countries experience a transition of the responsibility for public health in connection with cure, care, and welfare from national to local governments [45], which will make the local knowledge agenda more urgent. We therefore expect the demand from local governments for more socially robust scientific knowledge in public health to become stronger and that local governments will invest more in Mode-2 knowledge production. Research institutes should become more aware that researchers should identify, and deliberately seek to fill, policymakers’ knowledge gaps, to ultimately have any impact on the pull factors, which in the end will profit the research institute as well. Such awareness can be raised by a network formation of public health policymakers, practitioners, and researchers who regularly meet each other. They together create a context and a dialogue in which professional boundaries, political elements, and divisions in practice are much less emphasized, thus supporting the ‘blurring’ of boundaries [46]. For sustainability reasons, a mixed program of short- and long-term research is recommended to keep a balance in terms of push and pull factors. The challenge is to sufficiently blend both short- and long-term research within the organizational network with the ACC to make it practically impossible to separate them again.

Within the ACCs, partners should pro-actively negotiate and balance the push and pull factors and the accountability tensions. We also recommend that universities value both the scientific and societal impact of their research projects in a more balanced way [34]. Furthermore, the ACC coordinator needs to acquire a more balanced position in both the producer and user systems. The link with the user system, especially the policy domain, should therefore be strengthened. Exchange agents, e.g. the ACC coordinators, must yield to the researchers’ wishes and consent to the policymakers’ demands to keep the push and pull factors in balance with the performance indicators used by each institute. Coordinators can create a forum for the exchange of ideas between academics, policymakers, and practitioners to support the translation of knowledge from a research program and to articulate research questions based on problems in real-life policy and practice settings. There are no magic bullets, not one strategy fits all circumstances, but flexibility, boundary-spanning, negotiation, and familiarity with policy, practice, and research are relevant characteristics for the exchange agent’s role. Besides PHS appointed coordinators, the exchange agent’s role can also be expanded to coordinators appointed by municipality, thus creating a team to fulfil the exchange agent’s tasks, especially tasks related to the enabling pull factors.

Finally, to be able to meet future challenges in public health, we recommend that all relevant stakeholders at strategic positions from the domains of practice, policy, and research stimulate and facilitate the ACC networks to ultimately keep them sustainable and to realize the closer interactions between science and society.

## Abbreviations

ACC: Academic Collaborative Centres for Public Health
fte: full-time equivalent
PHS: Public Health Service
RCT: Randomized controlled trial
WHO: World Health Organization
ZonMw: The Netherlands Organization for Health Research and Development.
